# Exploring Reproducible Nonaqueous Scanning Droplet Cell Electrochemistry in Model Battery Chemistries

**DOI:** 10.1021/acs.chemmater.3c01768

**Published:** 2024-04-10

**Authors:** Alexey Sanin, Helge S. Stein

**Affiliations:** † Helmholtz Institute Ulm, Helmholtzstr. 11, 89081 Ulm, Germany; ‡ Karlsruhe Institute of Technology, 76021 Karlsruhe, Germany; § Technical University of Munich, TUM School of Natural Sciences, Department of Chemistry, Chair of Digital Catalysis; Munich Institute of Robotics and Machine Intelligence (MIRMI); Munich Data Science Institute (MDSI), Lichtenbergstr. 4, 85748 Garching b. München, Germany

## Abstract

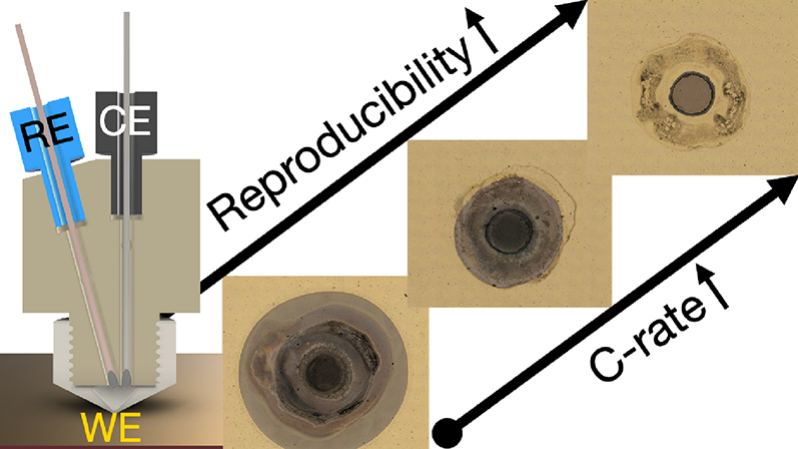

The discovery and optimization of new materials for energy storage are essential for a sustainable future. High-throughput experimentation (HTE) using a scanning droplet cell (SDC) is suitable for the rapid screening of prospective material candidates and effective variation of investigated parameters over a millimeter-scale area. Herein, we explore the transition and challenges for SDC electrochemistry from aqueous toward aprotic electrolytes and address pitfalls related to reproducibility in such high-throughput systems. Specifically, we explore whether reproducibilities comparable to those for millimeter half-cells are achievable on the millimeter half-cell level than for full cells. To study reproducibility in half-cells as a first screening step, this study explores the selection of appropriate cell components, such as reference electrodes (REs) and the use of masking techniques for working electrodes (WEs) to achieve consistent electrochemically active areas. Experimental results on a Li–Au model anode system show that SDC, coupled with a masking approach and subsequent optical microscopy, can mitigate issues related to electrolyte leakage and yield good reproducibility. The proposed methodologies and insights contribute to the advancement of high-throughput battery research, enabling the discovery and optimization of future battery materials with improved efficiency and efficacy.

## Introduction

With the world facing the challenge of climate change and greenhouse gas emissions reduction, it is important to have scalable and sustainable energy storage solutions for both stationary and portable applications to unleash the full potential of renewable energy sources and help the world transition to cleaner energy.^1^ Facilitating this transformation would require the implementation of advanced battery storage systems,^2^ making the discovery of new battery materials, as well as improving the existing ones even more critical than ever. The deployment of robotics and automation will enhance the efficiency and efficacy of battery research, while mitigating human error to meet the demands for high-performing energy storage materials. High-throughput experimentation allows the autonomous characterization of large numbers of samples in a relatively short time, which can speed up the discovery process and expand the scope of research.^3^ HTE techniques can apply to materials synthesis,^4^ crystal structure and chemical composition characterization,^5−7^ or cell assembly and cycling.^8,9^ Among the various HTE techniques for electrochemical characterization, scanning droplet cell and scanning electrochemical cell microscopy (SECCM) have a high potential for application in battery research. The major difference is that SDC can be used for materials investigation on a millimeter-scale, whereas SECCM is used on a micrometer-scale. The scale variation makes SDC a more versatile technique, allowing the elimination of particle orientation effects and providing information about average material properties, which is more relevant and closer to applied battery research.

SDC is a powerful tool for the electrochemical analysis in a sequential^10^ or parallel experimentation mode.^11^ Sequential experiments use a single cell whose tip is in close contact with the substrate. A small area under the tip is wetted by the electrolyte and takes part in the electrochemical measurement. Due to the local nature of the measurements, only a tiny amount of material is required for the measurement and there is no need to utilize any enclosures or separators, making materials discovery even more cost-effective.^12^ Instead of the conventional complex disassembly process of batteries, which requires specialized equipment, the disassembly and reassembly of an SDC-based battery experiment is achieved through automated motor movements and flow of the liquid electrolyte.

SDC has potential as a versatile tool for various applications in battery research for materials synthesis, electrolyte, and electrodes characterization. Rapid small-scale synthesis of thin film alloys using SDC by electrodeposition^13^ can be extended to oxides and polyanionic compounds. SDC enables rapid in situ formation and electrochemical characterization of solid electrolyte interphases (SEI) using redox mediators^14^ followed by subsequent ex-situ spectroscopic analysis by the analogy to the SECCM.^15^ It can also facilitate the optimization of electrolyte formulations, including those with additives, and the investigation of their conductivity and SEI properties.^14^ Moreover, SDC offers the potential for optimizing electrochemical processes in batteries, such as pulse charging.^16,17^ Combining flow-type of SDC setup with other techniques, such as mass spectrometry, enables detecting gas evolution, dissolution or degradation products during the electrochemical reactions in real time, providing valuable insights into the stability of electrode materials.^18,19^ High-throughput characterization of the materials libraries of potential battery anodes and cathodes materials could be done by the analogy as for investigation of materials for catalytic and photocatalytic activities.^10,20−22^

Although HTE using SDC is widespread in catalysis and corrosion studies,^21,23^ it has not yet been widely proliferated in the field of batteries. The reason is that many adaptations and optimizations are necessary, which are herein demonstrated and discussed. For example, conventional battery research should be carried out in an oxygen- and moisture-free atmosphere, which requires the device to be housed in a dry and inert glovebox. This adaptation has been successfully demonstrated in one of the few SDC publications for battery research by Dieckhöfer et al.^17^ However, some other reasons have not yet been discussed, such as the different physicochemical properties of the media. Catalysis and corrosion research using SDC is mostly performed in aqueous media where the measurement protocols and standard materials (e.g., droplet formation reproducibility and reference electrode (RE) materials) are well-established. In addition, the surface properties of catalysts, such as open circuit potential (OCP) or oxygen reduction or evolution reactions (ORR, OER) potential, are of the greatest interest and value to the scientific community, whereas in batteries both bulk and surface properties are important. The investigation of the bulk properties requires longer experimental times and the knowledge of extensive (scalable, e.g., mass of active material or electrochemically active area of the measurement point) parameters, which increases the probability of error and might additionally cause reproducibility and stability issues for multiple sequential measurements. The correct calculation of specific or areal capacity and energy from chronopotentiometry (CP), ionic conductivities from electrochemical impedance spectroscopy (EIS), or current densities from cyclic voltammetry (CV) requires a consistent reaction area within the experimental sequence.

Despite SDC appearing to have reproducible reaction areas within multiple measurements for aqueous systems,^11,24^ the reproducibility may not be guaranteed in nonaqueous media, which are commonly used in batteries. This has been previously shown in studies using SECCM.^25,26^ Additionally, one could also observe some deviations in the reaction area shape for lithium plating using SDC,^17^ though the discussion of this potential issue is omitted in the publication. However, there are some successful attempts to obtain the reproducible reaction area using SECCM with battery-grade electrolytes,^15,27^ and the reasons for such variation are not clear.

An alternative approach to overcome reproducibility issues is using masking, which has previously been used for aqueous-based corrosion research.^28−31^ During the mask preparation, a thin layer of inert material is applied on the surface of the investigated material, except the area of interest, making the reaction area more identical. Successful examples of such masks include photolithographic coatings,^28,29^ epoxy resins,^30^ and laser-drilled polymer tapes.^31^ Electrochemical experiments in nonaqueous electrolytes have, to the best of our knowledge, never been performed using those protective masks. The selection of the most suitable protective material and adhesive for battery research might be a challenging task since there is only little literature available regarding the chemical stability of different materials with battery-grade electrolytes.^32^ Still, the coupling of SDC and insulating masks could potentially be key for reproducible experimentation in the field of batteries.

Reproducibility testing for SDC in nonaqueous media is required to overcome the above-mentioned issues. In this article, we describe potential pitfalls and provide suggestions for improving nonaqueous Li-ion battery research using SDC. We present a facile and systematic procedure of how to select the most suitable REs, perform the reference measurement, and demonstrate the strategies with and without a masking approach to minimize reproducibility inconsistency. We believe that our research could offer valuable insights for the scientists employing SDC, SECCM, and other spatial electrochemical methods in nonaqueous media and could also contribute to the discussion of scientific experiments’ reproducibility.

## Methods

### SDC Setup

**Figure 1:**
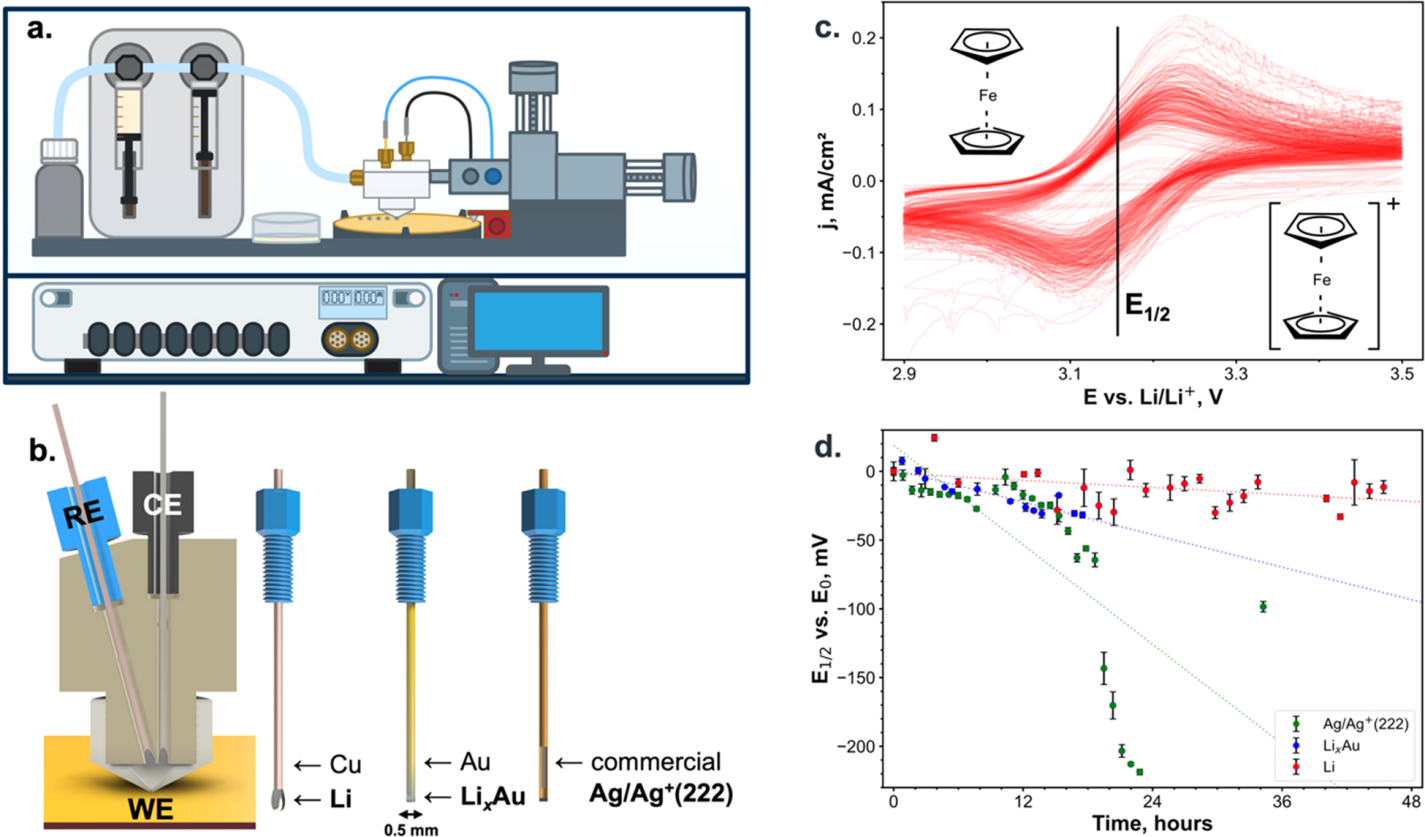
Schematic drawing of the SDC setup: the 3-electrode electrochemical cell with an opening is in contact with a substrate (WE), and a tight sealing is controlled by a force sensor. The electrolyte flow is regulated with a syringe pump. The coordinates of the cell are controlled by using high-precision stepper motors. All of the above-mentioned components are located inside the inert and dry argon glovebox. All the actions of the setup and electrochemical procedures are controlled using a PC and a potentiostat outside of the glovebox (a). The rendering of the scanning droplet cell cut in contact with Au substrate (WE), Li on Pt (CE), and Li on Cu (RE) is placed as close as possible to the tip opening without touching each other; the rendering of homemade lithium, lithium–gold, and commercial silver–ion (222) REs (b). 36 cyclic voltammograms for the reproducibility tests using Fc/Fc^+^ redox couple using Li RE. A half-wave potential is in the middle of the oxidation and reduction peaks (c). The half-wave potential Fc/Fc^+^ redox couple over the long-term experiment (up to 48 h) was measured using the various REs. The potential during the first measurement (*t* = 0 s) was considered as *E*_0_, and all other potentials were compared with this value (d).

The SDC setup consists of an electrochemical cell, a force sensor, high-precision motors, and a syringe pump (Figure 1a). The electrochemical cell is made of an inert polymer material (PTFE) with a cell volume of 55 μL (excluding the tubing) and a tip opening of 1.0 mm, which is in contact with the substrate during the experiment. Counter (CE) and reference electrodes are screwed into the cell body and located inside the cell as close as possible to the tip edge to minimize the uncompensated resistance (Figure 1b). In the SI there are technical drawings of the SDC head and tip (Figure S2). An electrolyte is dispensed or aspirated through the inlet from the side of the cell using a Hamilton syringe pump 700 with a 500 μL syringe (Hamilton Company, Switzerland). Before electrochemical procedures were started at a new measurement point, the cell is washed with a new portion of electrolyte (375 μL) to avoid cross-contamination across the materials library due to the residues from the previous experiments. The excess electrolyte is poured into the waste, and any electrolyte residues were removed from the PTFE tip. The position of the cell is controlled by high-precision linear stages and with stepper motors (OWIS, Germany). The tight sealing of the cell tip to the WE is controlled by a double bending force sensor KD45 (up to 2N force, ME-Systeme, Germany), and the force limit was set to 125 mN. All the above-mentioned components are housed in a MBraun UNIlab Pro glovebox filled with inert and dry argon gas (H_2_O and O_2_ content <1.0 ppm; MBraun, Germany). The setup is connected to an Autolab PGSTAT302N potentiostat galvanostat (Metrohm Autolab, Switzerland) and a PC outside of the glovebox, which orchestrates each command during the experiment through the HELAO framework using a Python code.^33^

### Materials Preparation

All electrochemical tests were performed with 1 M lithium hexafluorophosphate (LiPF_6_) in ethylene carbonate and ethyl methyl carbonate electrolyte (EC:EMC, 30:70 wt %) with 2 wt % vinylene carbonate (VC) additive (Elyte, Germany); for the REs test, ferrocene (≥99% purity, AlfaAesar, Germany) was added to the same electrolyte to obtain a 1 mM solution. The detailed description of WE, CE, and RE preparation procedures is available in the Supporting Information.

### Electrochemical Procedures

The OCP was measured for 300 s immediately after the contact with the substrate, for 300 s after CV and 600 s after the CP procedure.

CV with ferrocene-containing electrolyte was recorded at a sweep rate of 10 mV/s between 2.9 and 3.5 V vs Li/Li^+^, 2.4 and 3.2 V vs Li_*x*_Au, and −0.25 and 0.75 V vs Ag/Ag^+^(222) for 6 cycles. The half-wave potential of the Fc/Fc^+^ redox couple was calculated for each cycle, and then the mean and standard deviation were calculated from these statistics.

EIS was acquired at the OCP potential at the frequency range of 100 kHz (unmasked substrate) or 1 MHz (masked substrate) to 100 mHz with the 10 mV amplitude vs root-mean-square. The fitting of the spectra was performed using impedans.py Python package.^34^

CP was performed using a current density of 0.20 mA/cm^2^ between 0.01 (unmasked substrate) and 0.05 (masked substrate) and 1.00 V vs Li/Li^+^. The lower potential was chosen based on preliminary experiments to avoid lithium metal deposition. For a series of experiments with the current density variation, the current densities of 0.02, 0.05, 0.075, 0.10, 0.20, and 0.50 mA/cm^2^ were selected.

The OCP – CV – OCP – EIS – CP (discharge, charge) – OCP – EIS experiment sequence was used for long-term testing of the REs in a ferrocene-containing electrolyte with SDC. OCP was used for the equilibration of the electrochemical cell. CV measurements were used for RE potential measurement versus the Fc/Fc^+^ redox couple. EIS was used to track the changes in the electrode–electrolyte interphase. CP was used to analyze the dis-/charge behavior of the WE. For other reproducibility experiments with a standard electrolyte without ferrocene, OCP – EIS – CP (discharge, charge) – OCP – EIS sequence was used.

### Mass Loading and Thickness Measurements

The thickness and areal mass load of the sputtered thin film were analyzed using a HORIBA XGF-900 micro-X-ray Fluorescence Analytical Microscope (μ-XRF, Horiba Scientific, Japan). The XRF spectra were acquired at a minimum of 5 different spots on the substrate for 300 s using 50 kV X-ray energy (Rh source) and 100 μm polycapillary optics without an energy filter. The multilayer FPM function was used to determine the areal mass load amount and thickness of the thin films. Calculations were performed based on the intensity of the L_α_-line of Au and K_α_-lines of Cu and Si. Although μ-XRF is not a standard method for measuring areal mass loading in thin film research, for the specific objectives of our study, μ-XRF’s capabilities for evaluating areal mass loading were particularly relevant. Other standard methods include a quartz microbalance to estimate mass loading and X-ray reflectivity to estimate thin film thickness and density.

### Optical Microscopy

Prior to imaging, the substrate was rinsed 3 times with EC:EMC with a ratio of 30:70 wt % (Elyte, Germany) for 5 min followed by drying inside the argon glovebox for 15 min to remove the dried LiPF_6_ salt excess. Optical images of a SDC tip, perforated Kapton film and reaction area of the spots on a substrate were captured using a Keyence VHX 7000 optical microscope (Keyence, Germany) at 500 × magnification with the Stitching 3D Imaging option in HDR mode. To assess the reaction area of the working electrode the “maximum area” measurement function supplied by Keyence VHX software was used.

### Bootstrapping Procedure

For the bootstrapping procedure, 10000 bootstrapping samples were generated, each of which comprised 29 randomly selected data points, chosen with replacement (the same data point can have multiple occurrences in a single bootstrapped sample) from our original data sets of the specific capacities after each cycle for the unmasked and the masked substrates. For estimation of the minimal number of experiments, the number of randomly selected data points for the bootstrapping sampling procedure was varied.

## Results and Discussion

### Reference Electrode Selection

To select the optimal micro-RE with long-term stability, we conducted 36 experiments on 36 measurement points with a ferrocene-containing electrolyte (Figure 1c). The Fc/Fc^+^ redox couple is widely utilized as a reference redox system because its redox potential position is independent of organic solvents^35^ and is facile to be measured through CV analysis.^36^

Although silver-based REs are widely used for various battery chemistries,^37,38^ their reliability needs to be further improved for microelectrode setups. During the experiment, we observed unstable potentials after 18 h, which we believe to be induced by changes of the liquid-junction potential related to the RE filling solution being contaminated by electrolyte diffusion through the frit.^39^ Lithium–gold REs showed satisfactory performance with potential drifts within ±50 mV; however, the constant drift over time limits its usage for long-term experimentation. This behavior can be explained by the slow self-discharge of lithium-based alloys.^40^ We found that lithium REs have almost negligible drift, which makes them the most appropriate for the electrochemical measurements of large battery material libraries using a scanning droplet cell (Figure 1d). The reproducible behavior of the lithium REs was confirmed during another identical series of experiments over 132 h (Figure S3). Nevertheless, we would recommend performing the calibration of RE potential using a redox couple with a known potential before and after each experiment run. Despite the selection of the proper RE being crucial for the reproducible measurements, the cell geometry, electrolyte volume, electrode area, and position might also play a significant role in electrochemical potential drift.^41^

### Reproducibility Tests on Bare Au Substrate

For the reproducibility tests a gold thin-film substrate was selected as a standard WE because it can be used as a model anode material: it has already been successfully used to investigate SEI formation mechanisms and properties in lithium-ion batteries,^42,43^ but also its electrochemical activity and the ability to store lithium with alloying have been well studied.^44^ The lithiation process has two well-distinguished plateaus, making the alloying process more controllable and allowing to avoid the lithium metal plating; a wide voltage window and high electrical conductivity enable the usage of ferrocene as a reference redox couple; ease of thin-film preparation without any additional treatment; and the color change during the lithiation process promotes the control of the reaction area. Furthermore, the gained insights from the experiments with a gold anode can be used for understanding the lithium ions intercalation and deintercalation processes in other prospective negative electrode materials, which are suffering from extreme volume change, such as Si or Sn.^45,46^

To demonstrate the applicability of this method OCP, CV, EIS, and CP measurements were performed as these are commonly found electrochemical protocols in battery research.

**Figure 2:**
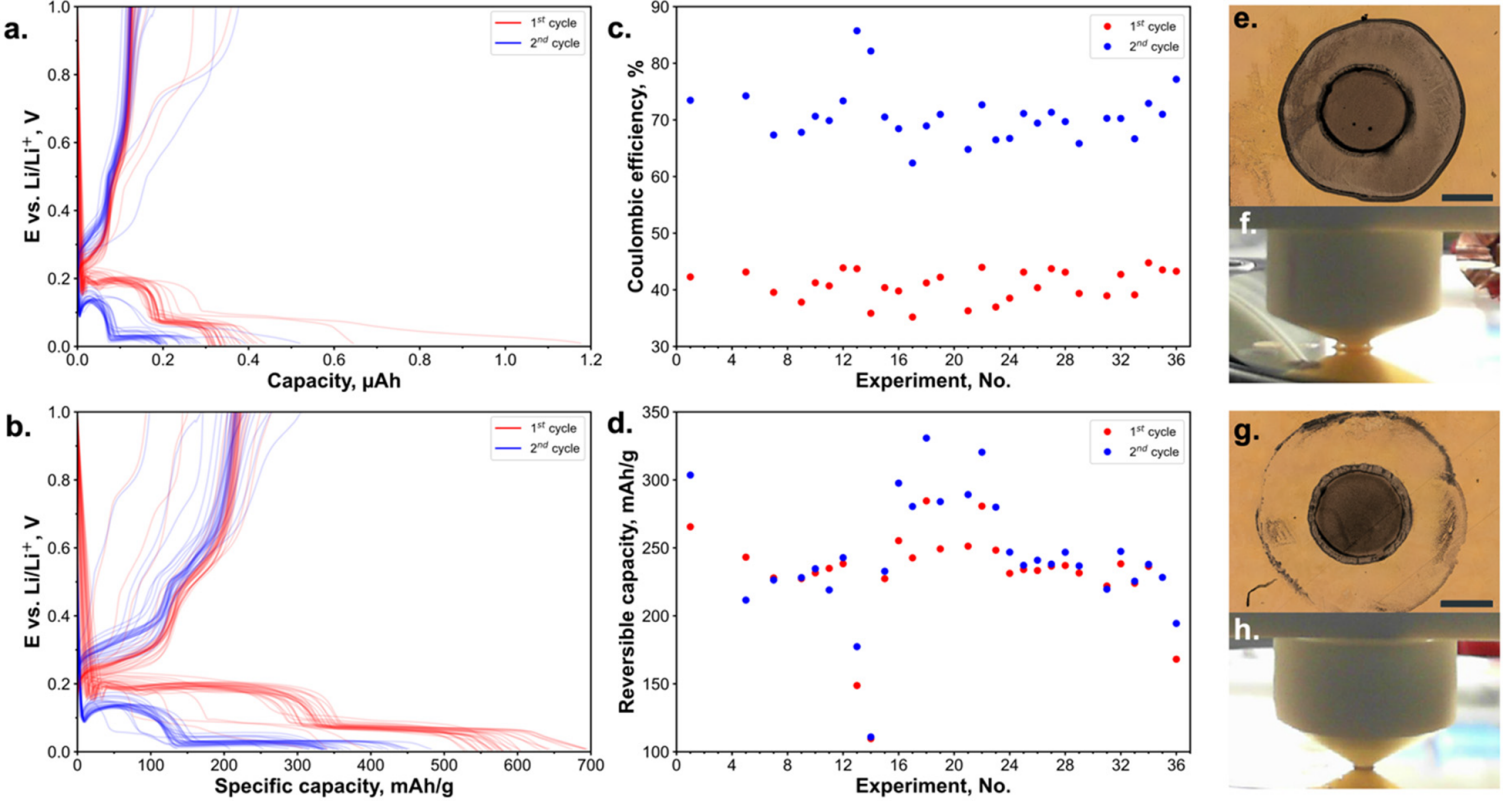
CP experiments on the unmasked substrate. Dis-/charge curves during 2 cycles for 36 experiments (a). The terms “discharge” and “charge” refer to the half-cell design of the SDC experiments. Dis-/charge curves during 2 cycles for 36 experiments; the specific capacity was calculated based on the reaction area from optical microscopy and areal load from XRF measurements (b). Coulombic efficiency for the 1st and 2nd cycles over 36 experiments (c). Reversible specific capacities for the 1st and 2nd cycles (d). The optical microscope images of the measurement spot: affected by the electrolyte leakage (e) and without a leakage (g); scale bars correspond to 500 μm. The photo of the electrochemical cell in contact with a substrate: with electrolyte leakage (f), without a leakage (h).

CP was used for discharging and charging a gold WE. The discharge profile shows a potential drop at the beginning of the charging process due to the alloy nucleation followed by two plateaus, and the charge profile includes three plateaus.^44^ The capacity loss during the cycling and low Coulombic efficiency might be attributed to the loss of active material due to extreme volume changes and SEI formation, which is commonly observed for alloy-type anodes.^45^ For the measurement on the unmasked substrate, most of the CP curves have identical shapes. However, some outliers in dis-/charged capacities were observed (Figure 2a), since the inconsistent contact between the cell and WE led to the leakage of the electrolyte around the tip and the formation of a meniscus (Figure 2f). Electrolyte leakage and spreading over the substrate can be explained by the relatively high surface energy of gold metal^47^ and lower surface tension of ester electrolyte solvents in comparison with aqueous systems.^48−50^ Optical microscopy images were acquired to investigate the leakage effect and to correct the reaction area. Although the majority of the measurement points displayed a reproducible reaction area (Figures 2g and S6), some spots had noticeable electrolyte leakage (Figures 2e and S6). Areas directly exposed to the electrolyte exhibit different colors from those under the sealing contact and the leakage area in the optical micrographs (Figures 2e and S4a). The variation between colors among these three areas might be attributed to the inhomogeneous lithiation and delithiation reactions, as well as SEI formation. The exact determination of the reaction area for some measurement points was a challenging task since the boundary between reacted and unreacted areas is not pronounced. The specific capacity values, corrected by the electrochemically active area and areal mass loading, exhibit less variance (Figure 2b,d). After this correction, the leaked spots demonstrate lower specific capacity, which could be due to changes in ionic conductivity caused by electrolyte evaporation in a leaked area, and incomplete discharge and charge processes resulting from the separation of leaked and nonleaked area by tip sealing. The Coulombic efficiency for the first and second cycles is consistent over all experiments (Figure 2c). However, even if the reaction area would be measured after the measurement, still the dis-/charge rate (in current per area or mass) would be different between measurement points, leading to additional deviations in the capacity values.

**Figure 3:**
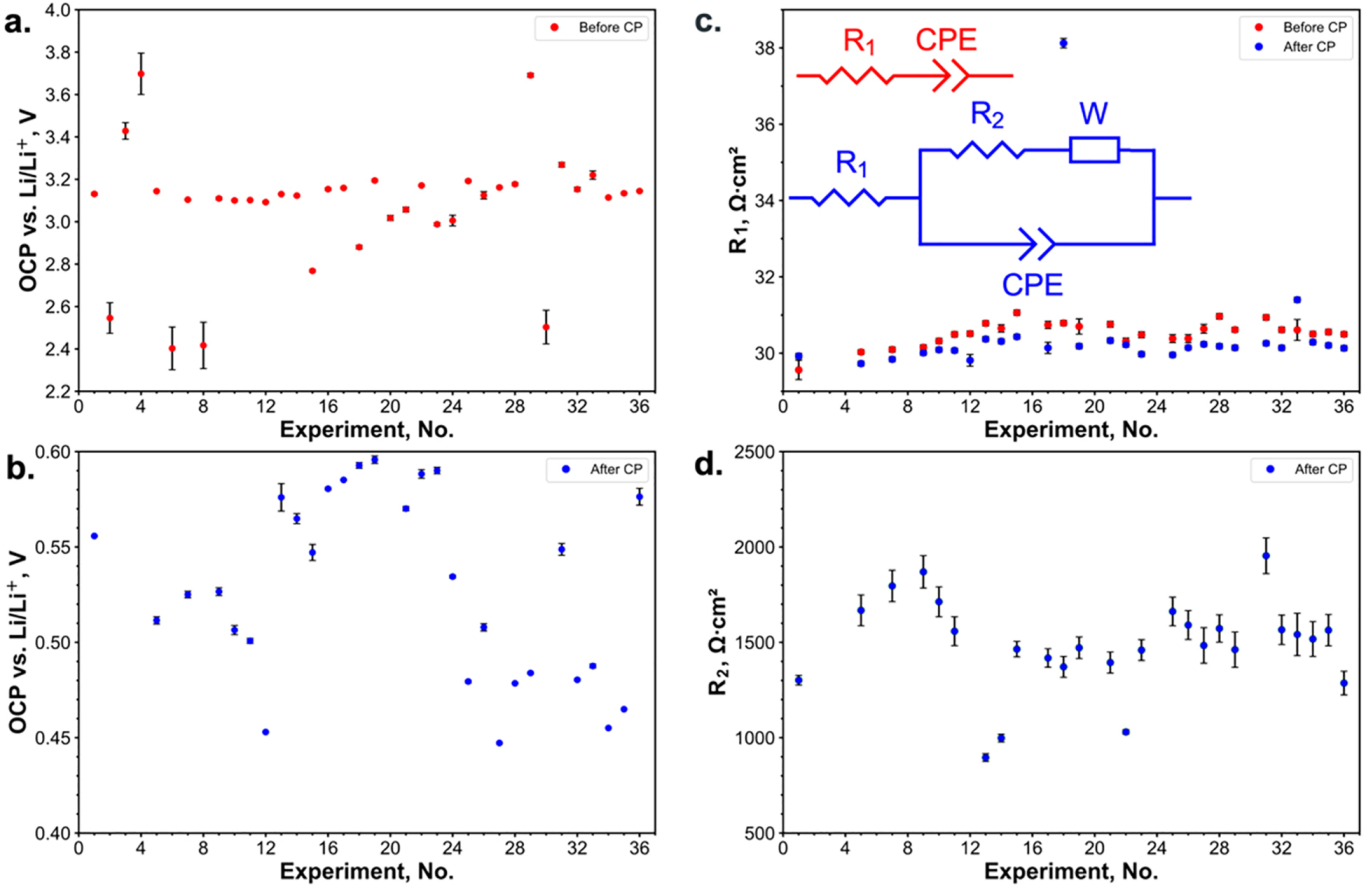
Results of the OCP and EIS experiments on the unmasked substrate. Average OCP of the last 120 s and 2σ-interval before (a) and after (b) the CP procedure. The equivalent circuits are represented for the measurements before (red) and after (blue) the CP procedure. Constant Phase Element (CPE) is indicative of the capacitive response, and the Warburg Impedance (W) corresponds to the mass diffusion response. R_1_ represents the combined contact and solution resistances before and after CP (c). R_2_ corresponds to the charge transfer and SEI resistance after CP (d). Areal resistances were calculated based on the SDC tip area.

The OCP procedure was used to equilibrate the electrochemical system after the contract with WE and before EIS. OCP measurement can be used as a quick method for poor contact detection between WE and electrolyte and potential stabilization before the EIS measurement. OCP was stabilized during ca. 1 min after the cell contact with the substrate and ca. 5 min after CP measurement (Figure S9a). OCP could also be used to detect RE drift (Figure 3a). The OCP lower than 1.0 V after the end of the second charge could be attributed to noncomplete delithiation^51^ (Figure 3b).

EIS was applied to monitor the WE interface evolution. At high frequencies, the uncompensated resistance is almost identical to the measurements taken before and after the CP procedure (Figure S10a). Despite the variability in the electrochemically active area, the high-frequency areal resistance (corrected by the tip area) is highly reproducible (Figure 3c). At lower frequencies, the difference is more pronounced, with an extra semicircle appearing in the measurement taken after the CP, which could correspond to the charge transfer resistance and electrical double layer and indicate the formation of the SEI (Figure S10b). The low-frequency resistance is less reproducible, with lower resistances observed for the spots with leakage, i.e., the larger electrochemically active area (Figure 3d). This discrepancy could be attributed to the fact that the signal at high frequencies is originating only from the exposed inner tip area. We thus assume that when there is leakage, the electrolyte trapped under the sealing area acts as a salt bridge that allows only for slow ion diffusion, i.e., similar to a thin capillary. This would explain the observed results similarity for the high-frequency impedance and differences for both low-frequency impedance and capacities. Therefore, extended-duration SDC measurements should be interpreted with caution in the presence of leakage. The EIS signal modeling should be performed, by the analogy to SECCM,^52^ to identify and verify the most suitable equivalent circuit describing electrochemical processes in this system.

### Effects of Current Densities on Leakage Behavior

**Figure 4:**
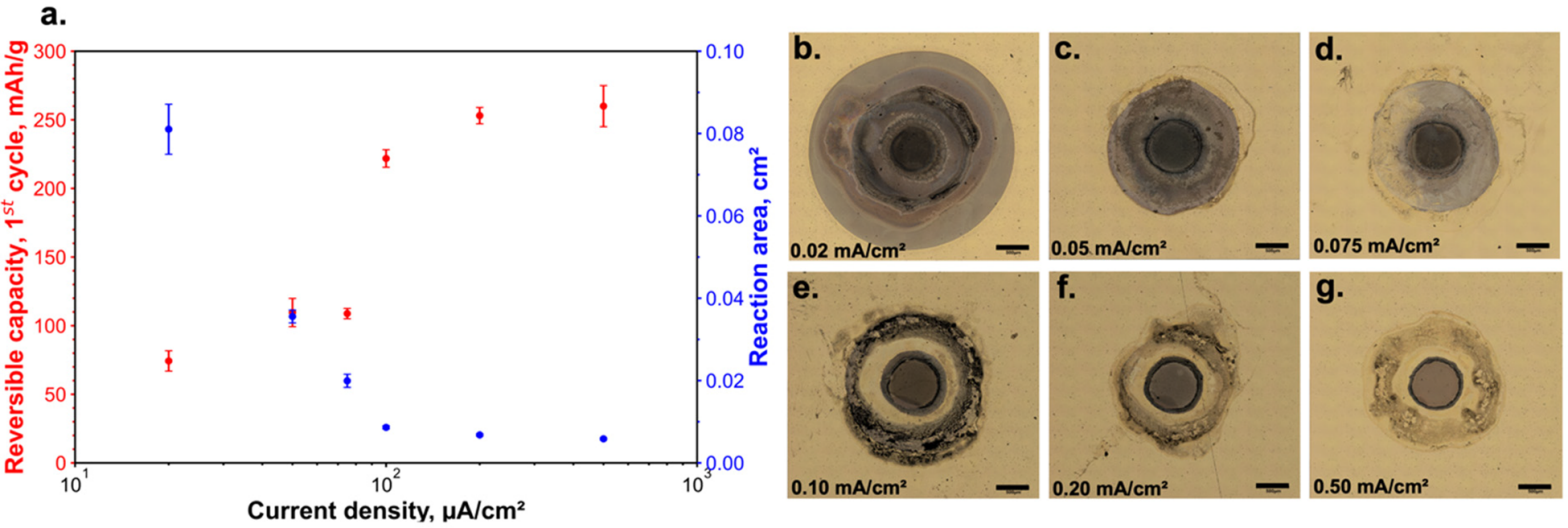
Reversible capacity after the first cycle and the reaction area as a function of the current density used to charge and discharge the electrode material. Current densities were calculated based on the tip opening, reversible capacity-based on the reaction areas from microphotographs (a). The optical microscope images of the measurement spots for different applied current densities (b–g); all scale bars correspond to 500 μm.

To further understand the variables affecting leakage behavior in electrochemical measurements, we conducted a series of experiments on an unmasked substrate with varying dis-/charge currents. Our data reveals a notable correlation between the applied current density and the measured reaction area (see Figure 4). We observed that the longer the electrochemical measurement is performed on a measurement spot, the higher the observed probability of electrolyte leakage and the larger the electrochemically active area. We identified a critical current density threshold of 0.075 mAh/cm^2^, above which leakage is significantly reduced in our case study. The gray color of the outer ring for the sample with the used current density of 0.075 mAh/cm^2^ and low reversible capacity (Figure 4d,a) indicates that the lithiation reaction of gold in this region is only partial.

This leakage behavior has not been previously observed in shorter-duration catalysis or corrosion studies in aqueous media using SDC. Based on these findings, we recommend rapid electrochemical tests, involving higher current densities within a short duration to effectively minimize electrolyte leakage and improve reproducibility. Therefore, in battery research, SDC holds the greatest promise in the exploration of fast charging capable materials.

### Reproducibility Tests with Masking

Surface masking with polymer films, which have lower surface energy properties than metals or oxides,^53^ could potentially not only reduce the electrolyte leakage, but also control the reaction area more precisely. Kapton (polyimide) film was chosen as a masking layer due to its excellent electrical insulation of protected areas and relatively good chemical resistance to battery-graded electrolytes, as it has been used successfully in in situ X-ray diffraction experiments for batteries.^54^

**Figure 5:**
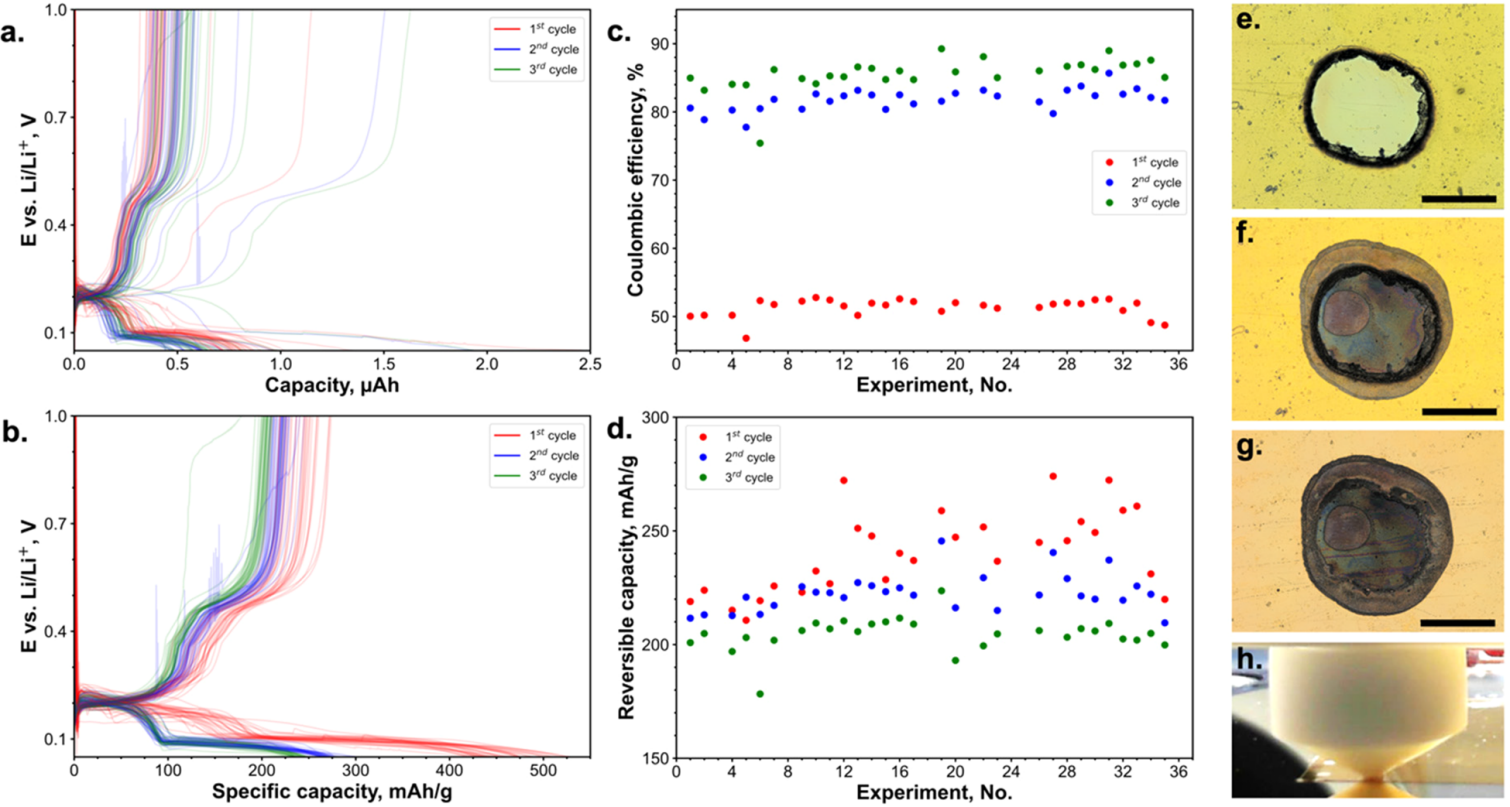
CP experiments on the masked substrate. Dis-/charge curves during 3 cycles for 36 experiments (a). Dis-/charge curves during 3 cycles for 36 experiments, the specific capacity was calculated based on the reaction area from optical microscopy and the areal load from XRF measurements (b). Coulombic efficiencies for the 1st, 2nd, and 3rd cycles over 36 experiments (c). Reversible specific capacities for the 1st, 2nd, and 3rd cycles (d). The optical microscope images: a spot with perforated Kapton film on Au before electrochemical experiment (e), a spot with perforated Kapton film on Au after electrochemical experiment (f), a spot on Au after electrochemical experiment after Kapton film removal (g); the scale bars correspond to 500 μm, and the dark spots on figures (f) and (g) correspond to the reaction area. The photo of the electrochemical cell in contact with the masked substrate (h).

The significantly larger size of the reaction area compared to the perforated area (Figure 5f,g) might be attributed to crevice corrosion of the film adhesive as the laser burns a film and adhesive at the edge of the perforation (Figure 5f). After removal of the mask, partial delamination of the gold thin film was observed at the adhesive contact area. Three dark encirclements are visible, each attributed to the edges of the reaction area at the end of the lithiation process in each of the three cycles (Figures S4b and S7). This implies that some leakage might be unavoidable over time due to electrowetting during galvanostatic measurements.^55^ Despite variations in the measured area, the applied areal correction shows high reproducibility of the CP measurements, as evidenced by the notable overlap of discharge and charge curves as well as the consistent values of reversible specific capacity (Figure 5b,d). In contrast, for EIS measurements, the inconsistency before and after can be attributed to both high- and low-frequency resistances; with no correlation observed between the resistances and perforated area or reaction area (Figure S10c,d), this might be interpreted by different signal contributions from the perforated and leaked area of the mask.

### Statistical Variation

#### Between the Measurements on One Substrate

Out of 36 experiments, 29 (80.5%) are successful for both the unmasked and masked substrates, respectively. The failures mostly stem from poor contact (Figures S5, S6, S7, and S8), so it is crucial to find the optimum between the leakage of electrolyte and contact loss, by varying the dispensing and aspiration procedure of the extra portion of electrolyte before or after the contact with the substrate. The implementation of the algorithms that could decide the quality of the electrolyte contact with the cell based on OCP (Figure 3a) or EIS (Figure 3c,d) measurements and pump an additional amount of electrolyte could also be useful to detect and overcome this issue, and also to improve the output of the research. All specific reversible capacities have a 95% confidence interval of less than 5.5% of the mean (Table S1), and for the second cycle, the margin of error is only 3.3% and 1.4% for the unmasked and masked substrates, respectively. The uncertainties for the Coulombic efficiencies are less than 1.3% and for the OCP measurements are less than 60 mV before the CP procedure and less than 20 mV for the measurements after the CP procedure. Since the presence of lithium-ions is expected for both the electrolyte and WE after the cycling, more reliable measurements of the electrochemical potential are expected. The margin of error of resistive elements for a substrate without a mask is less than 1.9% for the high-frequency resistance and 10.4% for the low-frequency resistance, which is probably related to the different signal contributions of the leaky and nonleaky areas. The resistances are within 9–75 percentage points of the real population value 95% of the time for the masked substrate.

#### Between Two Substrates

The reversible specific capacities are comparable to those without masking (the means are 230.6 and 240.6 mAh/g for the first cycle and 243.7 and 222.6 mAh/g for the second cycle), considering the uncertainty of the thickness measurements, variation in effective current densities, and different cutoff potential. The Coulombic efficiencies are reproducible between different spots, and better for a thicker substrate, which might be attributed to the lower surface area to lower effective current density (corrected by the leakage area) or volume ratio of the thin films.^56^ The difference in the OCP potentials is less than 150 mV for the measurements before dis-/charge cycles and less than 20 mV after cycling. EIS results are hard to compare since of the difference in the geometry of leaked areas and since the resistances are also dependent on the position of the RE, which might change between the two series of experiments.

#### Between Other Cell Geometries

**Figure 6:**
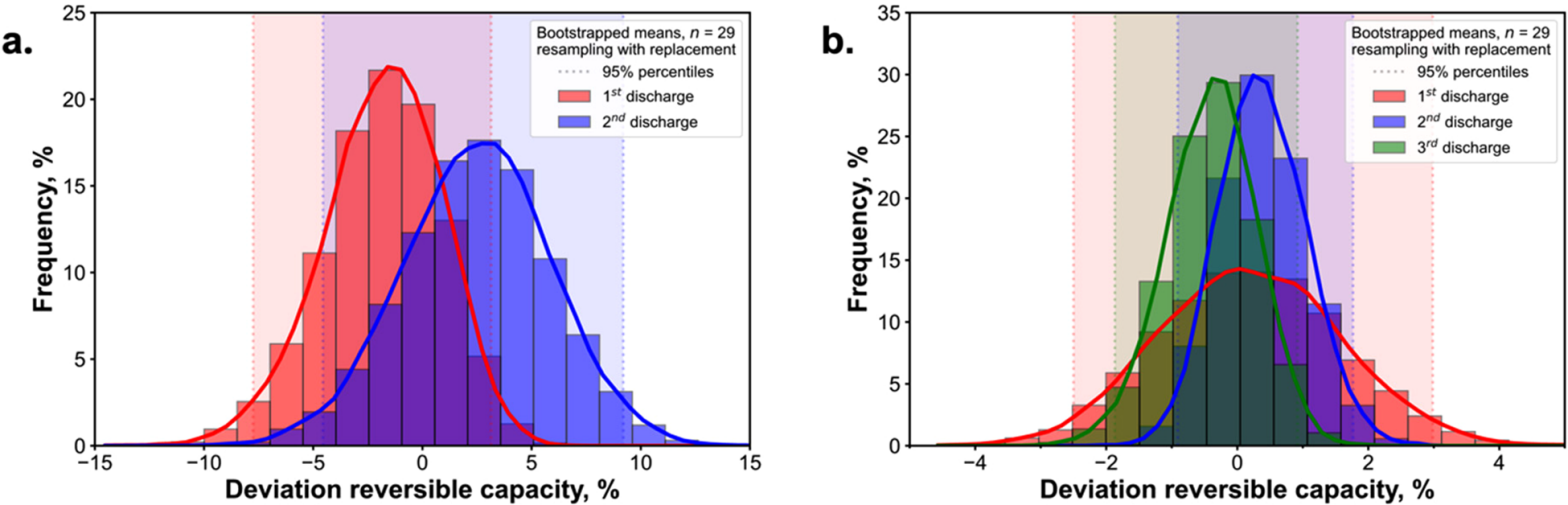
Bootstrapping statistical analysis: the distribution of reversible capacity deviation from the median and the 95th percentiles for the respective cycle for the experiments on the unmasked substrate (a) and the masked substrate (b).

The bootstrapping procedure was employed to assess the experimental variability and to estimate the whole population of values. By generating a larger data set, this approach gives a detailed representation of the underlying population distribution. It also provides a “statistically bootstrapped” estimate of our data trends that is less susceptible to the influence of outliers and inliers (i.e., noise below a threshold) or sample size variability. The mean reversible specific capacity during the third cycle on the masked substrate, as determined from the bootstrapping, is 204.5 mAh/g with 95% percentiles of (201.6, 207.1) mAh/g, corresponding to a maximum 1.4% margin of error. The relative standard deviation from the bootstrapping analysis is 0.7%, which is significantly superior to the results previously obtained in our laboratory for the cycling of coin cells assembled using the robotic system.^57^ However, the capacity values for the robot-assembled coin cells were not normalized by mass and had a bootstrapped bimodal distribution, while for our system, a Gaussian-shape distribution is observed (Figure 6). To estimate the minimal number of required experiments to understand the interexperimental variability, the sampling procedure followed by bootstrapping could be applied, and the uncertainty threshold should be specified based on the goals and reproducibility criteria of the research. We suggest that 8 or 9 SDC experiments are an easily accessible lower threshold for the minimum number of repeats (Figures S12, S13), comparable to those reported by Dechent et al.^58^

## Conclusions

This research represents high-throughput experimentation using SDC for nonaqueous lithium-ion battery systems, including a masking approach to address the challenge of maintaining good and reproducible contact between the electrochemical cell and substrate without electrolyte leakage. Our findings indicate the reliability of SDC for electrochemical testing of battery materials, which can provide results on par with or better than those for automatically assembled coin cells. Nevertheless, we acknowledge that further advancements are still required to completely prevent electrolyte leakage.

The coupling of SDC measurements with optical microscopy to determine the reaction area has the potential to mitigate measurement deviations of electrolyte leakage and improve the data reproducibility. Our results showing that electrolyte leakage is dependent on current density or experiment duration highlight the need for careful experimental design. We suggest utilizing the masking approach for long-time experiments and analysis of extensive system parameters, while for short-time experiments and intensive figures of merit, the procedure without a mask would be the most effective.

We propose using a gold thin film as a standard material for system calibration before running the actual experiment and lithium metal as a RE for nonaqueous lithium-ion materials research with SDC. The protocol demonstrated in this paper could be tailored for selecting alternative REs for postlithium chemistries. The demonstrated reproducibility suggests that SDC experiments can yield consistent data whenever the intensive properties are of interest, which are independent of the reaction area or material amount, and when the extensive properties such as capacity are of interest, additional means of verification such as optical microscopy should be utilized.

## Supporting Information Available

Electrochemical data (HDF5) (ZIP)
Video of 24 h SDC operation during the sequential experiment (MP4)
Detailed experimental procedures of the SDC body and tip, WE, CE, and RE preparation, the technical drawing of the SDC body and tip, reference electrode stability data, optical microscopy data, OCP and EIS data, EIS equivalent circuits and fitting data, and bootstrapping analysis data (PDF)
