# Supplementary material for: Exploring Reproducible Nonaqueous Scanning Droplet Cell Electrochemistry in Model Battery Chemistries
Source: Chem Mater. 2024 Apr 10;36(8):3536–45. doi: 10.1021/acs.chemmater.3c01768 (PMC11044270; doi:10.1021/acs.chemmater.3c01768)
Supplement: Supplementary file 3 — cm3c01768_si_003.pdf [file cm3c01768_si_003.pdf]

Supporting information

Exploring reproducible non-aqueous scanning droplet cell electrochemistry in model battery chemistries

Alexey Sanin[1,2,*], Helge S. Stein[1,2,3]*

1: Helmholtz Institute Ulm, Helmholtzstr. 11, 89081 Ulm, Germany
2: Karlsruhe Institute of Technology, Institute of Physical Chemistry, Fritz-Haber-Weg 2, 76131 Karlsruhe, Germany
3: Technical University of Munich, Department of Chemistry, Lichtenbergstr. 4, 85748 Garching b. München, Germany
*Correspondence should be addressed to: alexey.sanin@kit.edu, helge.stein@tum.de

Gold thin-film substrate preparation

WE substrates were prepared by reactive magnetron sputtering in a sputtering chamber (Creamet 400 multi3, Creavac, Germany) inside an argon glovebox (< 0.1 ppm H_2O, < 3 ppm O_2; SylaTech, Germany). An RF bias (P = 75 W, t = 600 s, p(Ar) = $6.1\cdot10^{-3}$ mbar, at room temperature (RT)), was applied to a Si wafer (CZ-Si wafer, diameter = 4”, thickness = 525±25 µm, (100), 1-side polished, p-type (Boron), 1-10 Ohm, MicroChemicals, Germany) to clean the surface before the sputtering of the metals. A copper thin film was sputtered for 5 min as a backing layer and current collector from the Cu target (100 mm diameter, 5 mm thickness, 4N purity, EvoChem, Germany) at P = 200 W direct current (DC), p(Ar) = $4.3\cdot10^{-3}$ mbar, RT; then a gold thin film was sputtered from the Au target (50 mm diameter, 5 mm thickness, 4N purity, EvoChem, Germany) at P = 50 W DC, p(Ar) = $6.5\cdot10^{-3}$ mbar, RT. During the sputtering process, the substrate holder was rotated to achieve uniform thickness of the thin film over the entire substrate. All the materials targets were pre-sputtered for at least 1 min to avoid any contamination. Thin films were sputtered for different times (4 min for an unmasked sputtering and 10 min for a masked substrate) to obtain WEs with different thicknesses to enable reproducibility tests of specific capacity.

For the experiments with Kapton masks, a single-sided Kapton polyimide tape with silicone adhesive (60 µm thick, Micro to Nano, the Netherlands) was perforated using a Mr. Beam II Dreamcut laser (Mr. Beam, Germany), the desired perforation diameter was 0.5 mm. The perforated film was wiped with isopropanol to remove burnt residues and transferred to a Si wafer with a gold-sputtered thin film.

Electrodes preparation

Before the experiments, Pt (99.997% (metal basis), 0.5 mm dia., Premion®, AlfaAesar, Germany), Au (99.9985% (metal basis), 0.5 mm dia., Premion®, AlfaAesar, Germany) and Cu (99.999% (metal basis), 0.5 mm dia., Puratronic®, Thermo Scientific, Germany) wires were glued into a PEEK screw-nut, additionally polished with a 3M™ Paper Sheet 255P sandpaper and then cleaned. The Pt and Au wires were cleaned for 5 min in a hot Pirahna solution of H_2SO_4 (95-97%, Supelco, Merck, Germany) and H_2O_2 (30%, Perhydrol®, Supelco, Merck, Germany), mixed in a 3:1 ratio. Then, the wires were rinsed with deionized water (resistance of 18.2 MΩ·cm, from Milli-Q™ Reference Ultrapure Water Purification System, Merck, Germany) and immersed in the ultrasonic bath with deionized water for 15 min and isopropanol

(≥98%, VWR Chemicals, Germany) for additional 15 min to remove any acidic traces. Finally, the wires were dried at 80°C for 12 h and transferred to the argon glovebox.

For the preparation of a Li on Pt CE, lithium metal (99.9% trace metal basis, Sigma-Aldrich, Germany) was melted in a stainless-steel crucible at ca. 250°C inside the argon glovebox (H_2O and O_2 content < 1.0 ppm; MBraun, Germany), then a Pt wire was dipped into the molten lithium to form a drop of lithium metal at the edge of the wire. The identical procedure was used to prepare Li RE, where Li drop was placed on Cu wire. A lithium-gold RE was prepared in-situ in a scanning droplet cell. During the electrochemical procedure SDC was in contact with a lithium ribbon, which served as a lithium source and CE, gold wire served as the WE, Li on Cu wire – as the RE (Figure S1). For a lithium-gold alloy formation, a current of -5 µA was applied for 3600 s. For the comparison, a commercial micro-RE (Ag // 0.01 M $AgNO_3$, 0.01 M cryptand-222, 0.5 M Bu_4NPF_6 in MeCN from rhd instruments, Germany) was used (Figure 1b). Prior to the experiments, Li and Li_xAu REs were soaked in an electrolyte solution for at least 1 h, while $Ag/Ag^+(222)$ was soaked for 12 h to equilibrate of the potential. A Li-coated Pt spiral wire used as a CE was prepared using the same procedure as Li REs.

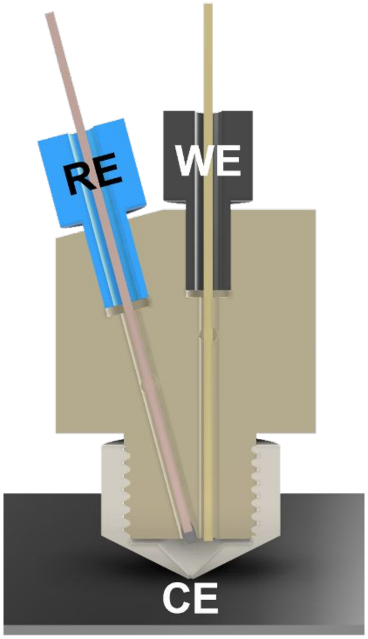

Figure S1. The SDC setup for in-situ preparation of Li_xAu reference electrode: during the lithiation process, the Au wire is used as a WE, Li ribbon is used as a CE and Li on Cu wire as a RE.

SDC body and tip preparation

The PTFE SDC body (technical drawing is shown in Figure S2) and tip (Sensolytics GmbH, Germany) were cleaned in the same way as the Pt and Au wires. After assembling the SDC cell, a tip was screwed onto the SDC body and then polished with the 0.3 µm polishing paper (Sensolytics GmbH, Germany) using acetonitrile (≥99.5%, for HPLC, WVR Chemicals) as a solvent for cleaning of the SDC tip. The applied force during the contact of SDC head and the polishing paper was between 100 and 125 mN.

Figure S2. The technical drawing of SDC cell body and tip

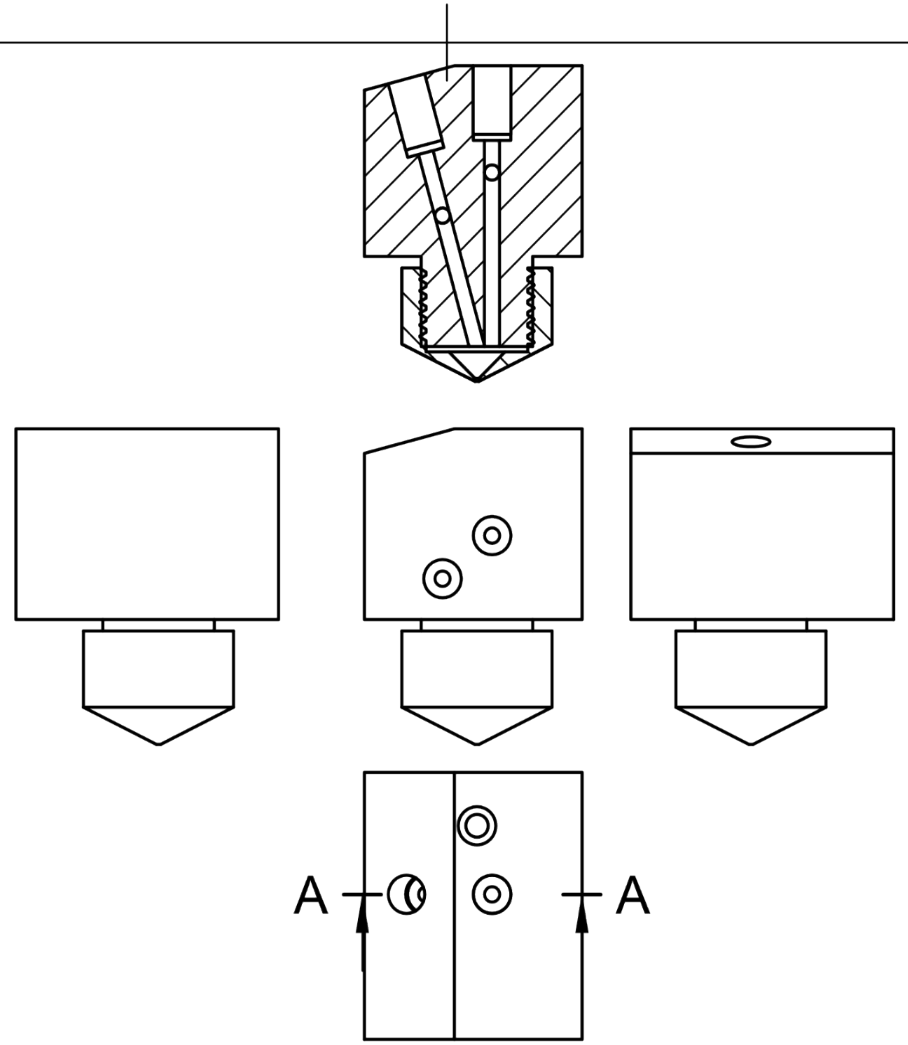

Dept.	Technical reference	Created by	Approved by
		Helge Stein 13.06.23	
		Document type	Document status
		Title	DWG No.
		ptfe_SDC_body	
			Rev. | Date of issue | Sheet
			1/1

Lithium reference electrode stability tests

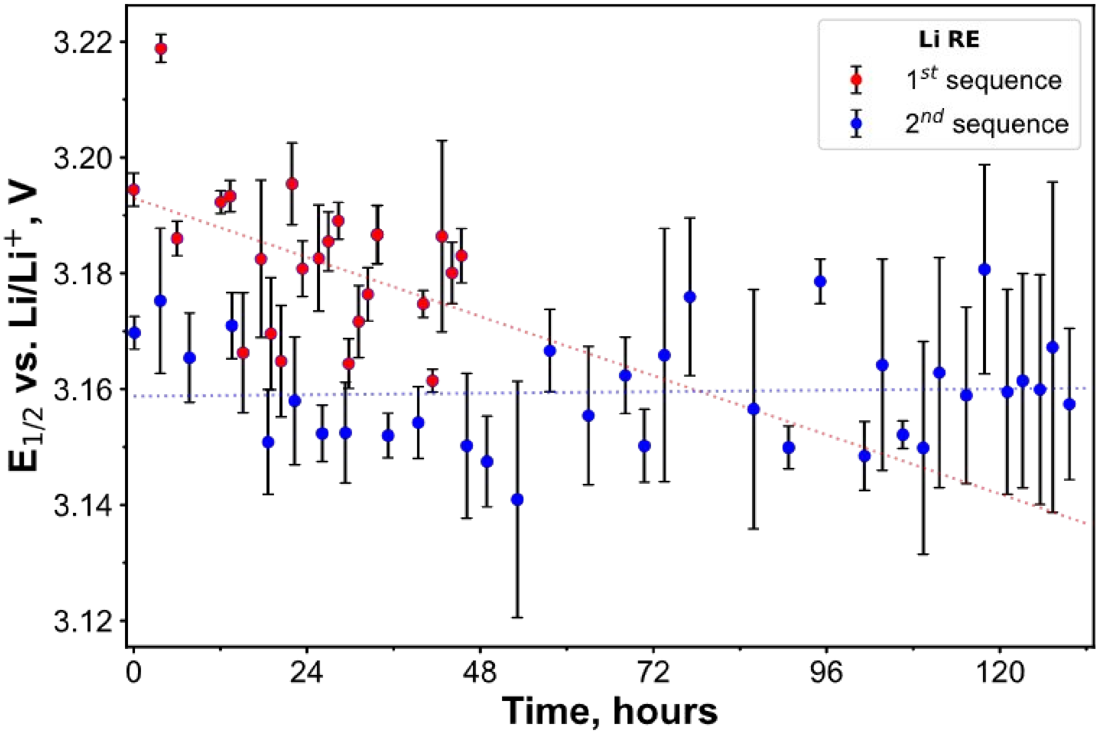

Figure S3. The half-wave potential Fc/Fc$^+$ redox couple over two long-term experiments (up to 48 and 132 h) was measured using a lithium reference electrode.

Optical images of wetted areas

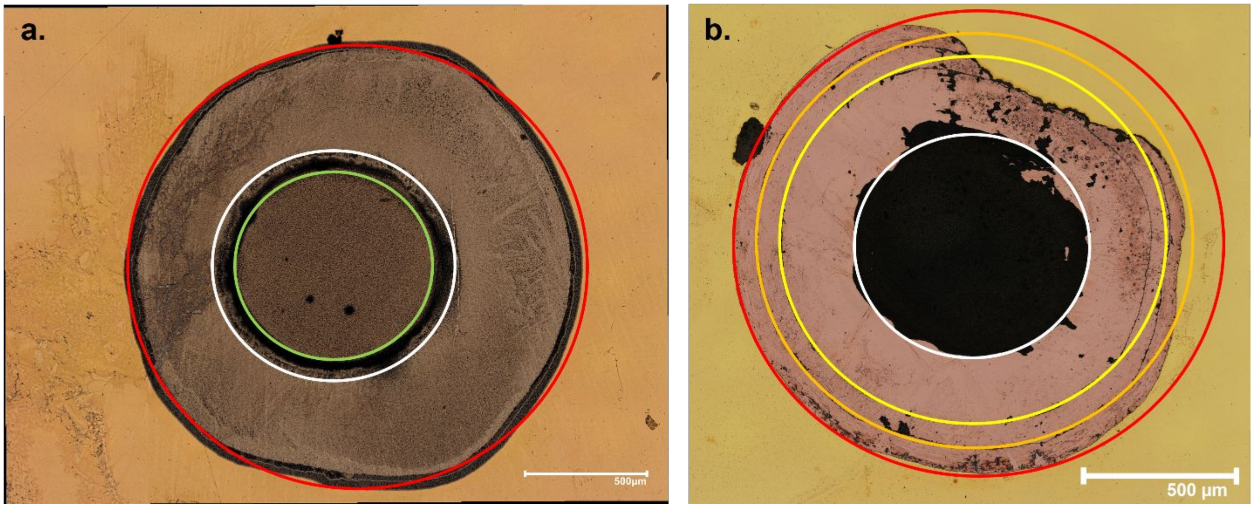

Figure S4. An example of wetted area regions after the experiment for a substrate without a mask (a): wetted area under the inner tip area (green encirclement), the wetted area under the tip sealing area (white), and the area of electrolyte leakage (red). An example of wetted area regions after the experiment for a substrate with a Kapton film mask (b): During the removal of the Kapton film a part of the reacted gold thin film was delaminated, so Cu backing layer is observed. The non-laminated area is within the white encirclement. Yellow, orange, and red encirclements correspond to the wetted area after the 1st, 2nd and 3rd cycles.

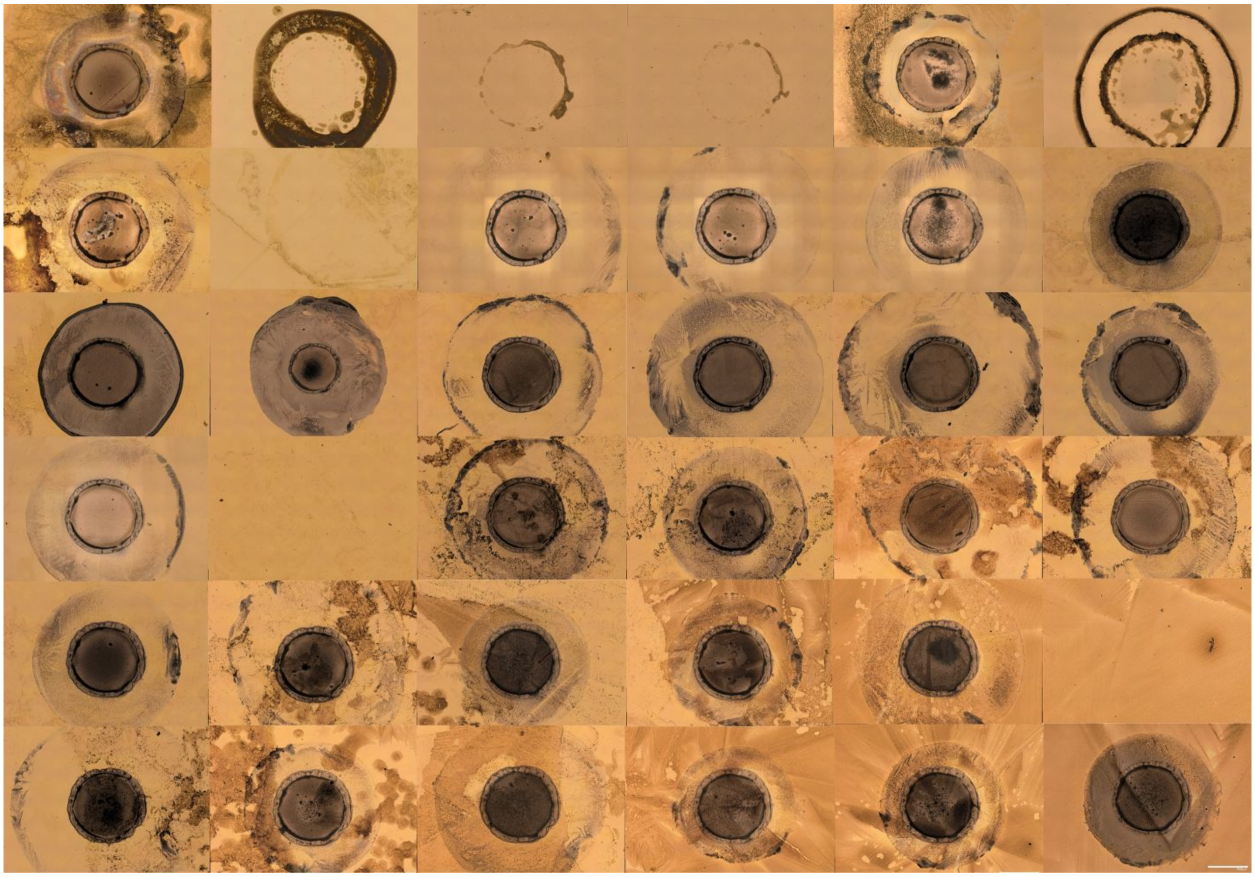

Figure S5. The optical images of 36 measurement spots on the unmasked substrate after the end of the experiment sequence. The white bar on the right bottom image corresponds to 500 µm.

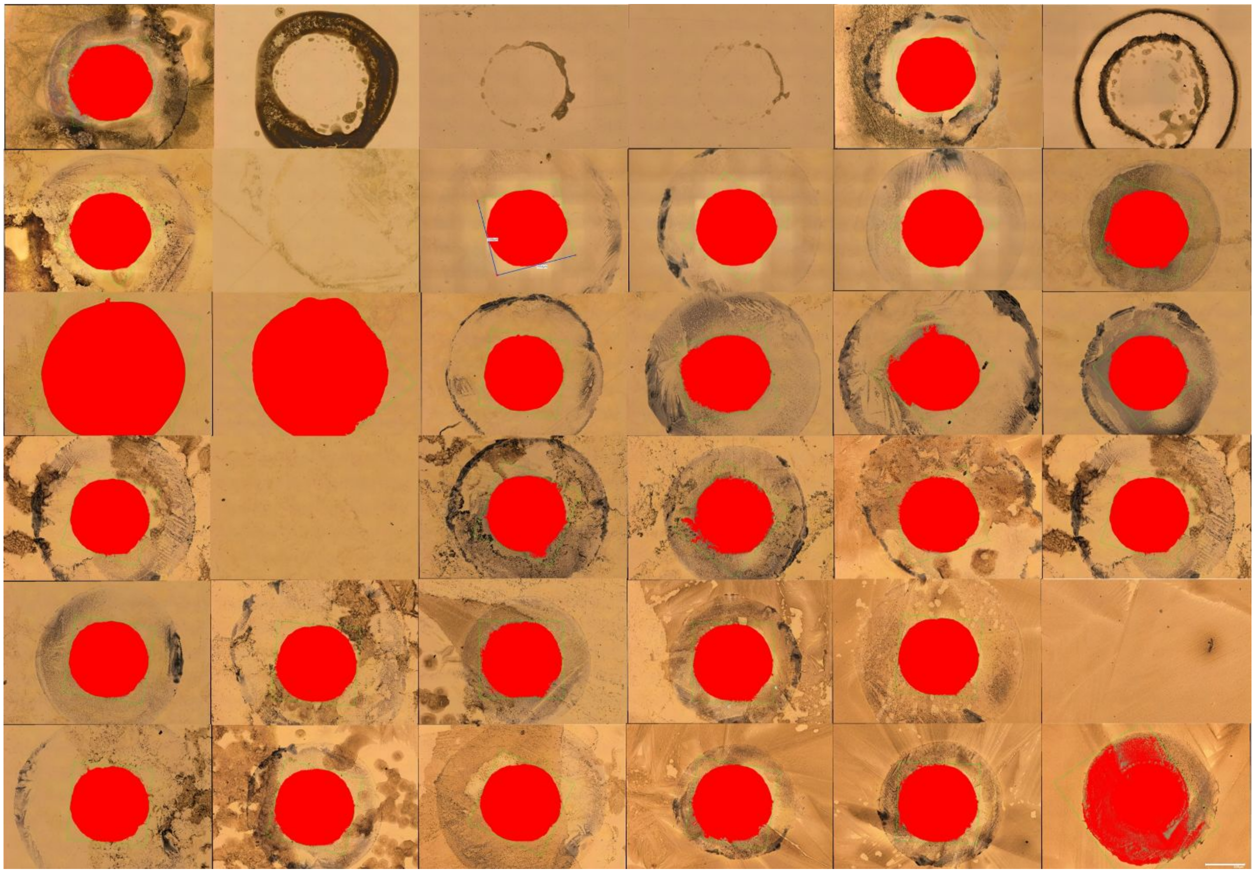

Figure S6. The optical images of 36 measurement spots on the unmasked substrate after the end of the experiment sequence, red-colored areas correspond to the wetted area based on the colour difference. The white bar on the right bottom image corresponds to 500 µm.

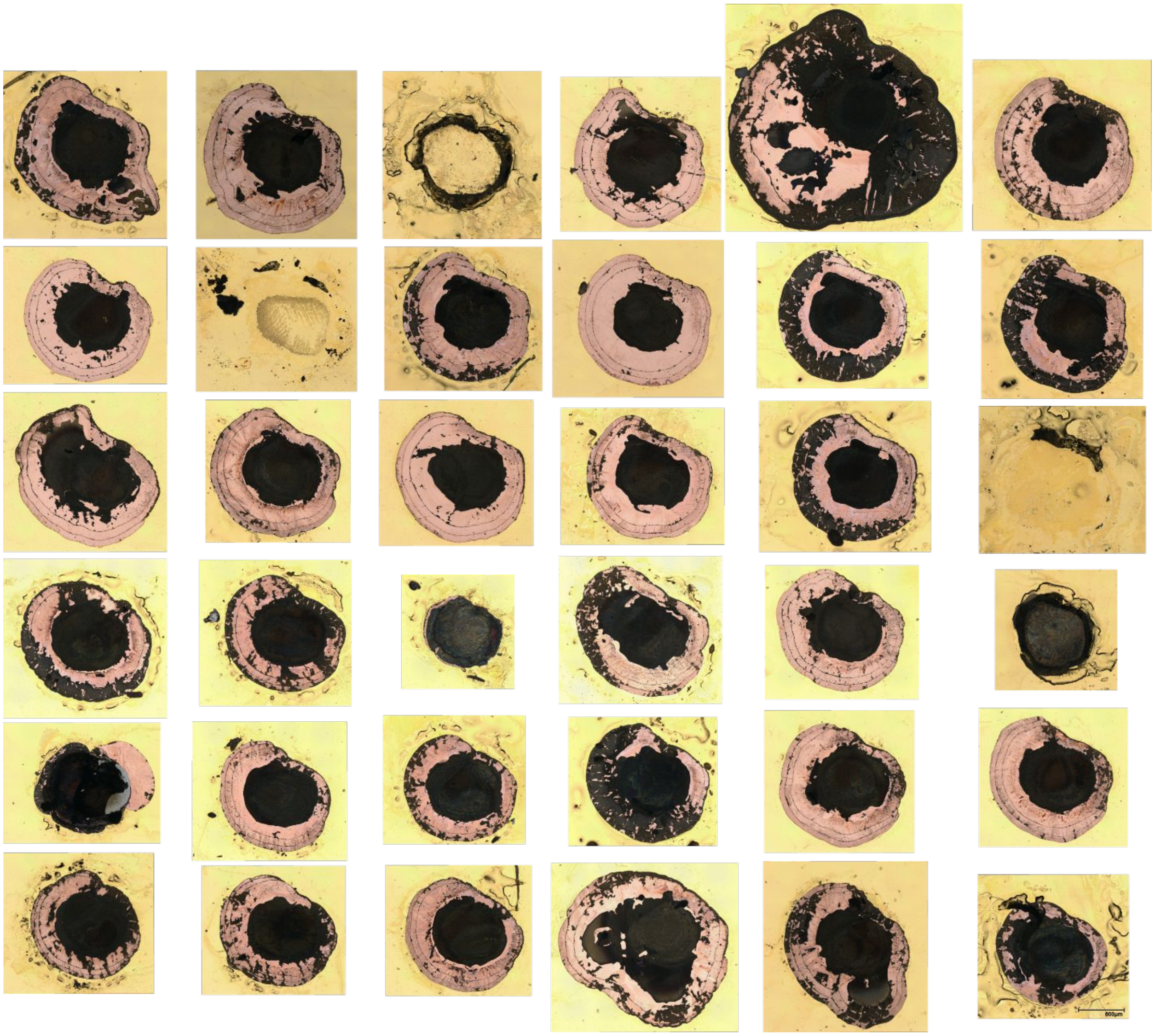

Figure S7. The optical images of 36 measurement spots on the masked substrate after the end of the experiment sequence. The pink-red area shows where the lithium-gold alloy film was delaminated after the removal of the Kapton film, exposing the copper current collector. The middle black area was not delaminated because it was not covered by Kapton film. The black bar on the right bottom image corresponds to 500 µm. All the images are shown on the same scale.

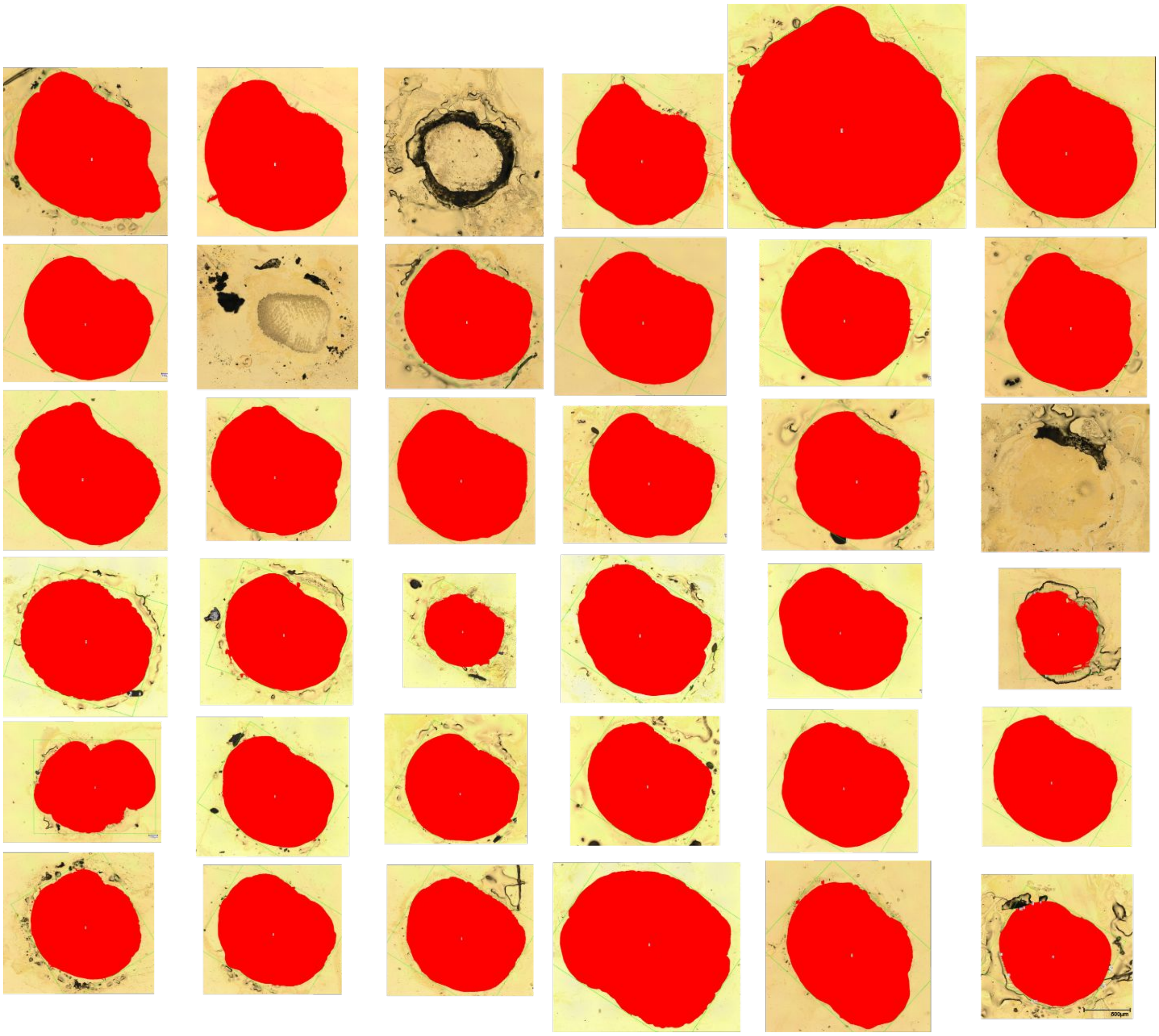

Figure S8. The optical images of 36 measurement spots on the masked substrate after the end of the experiment sequence, red-colored areas correspond to the wetted area based on the colour difference. The black bar on the right bottom image corresponds to 500 μm. All the images are shown on the same scale.

Open circuit potential measurements

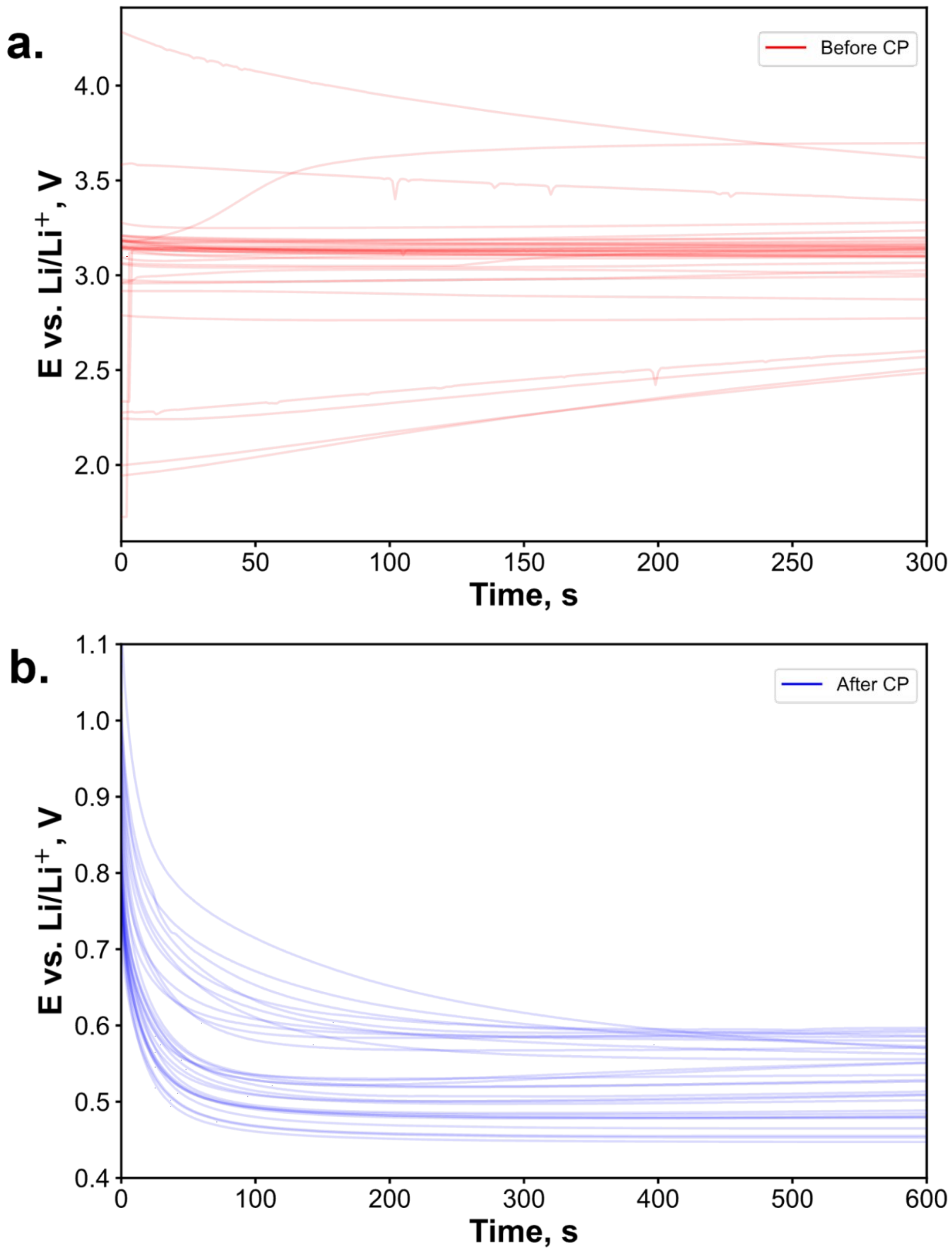

Figure S9. Open circuit potential measurements on 36 different spots on a substrate without masking: directly after the SDC contact with the substrate before CP measurement (a), after CP measurements (b).

Electrochemical impedance spectroscopy

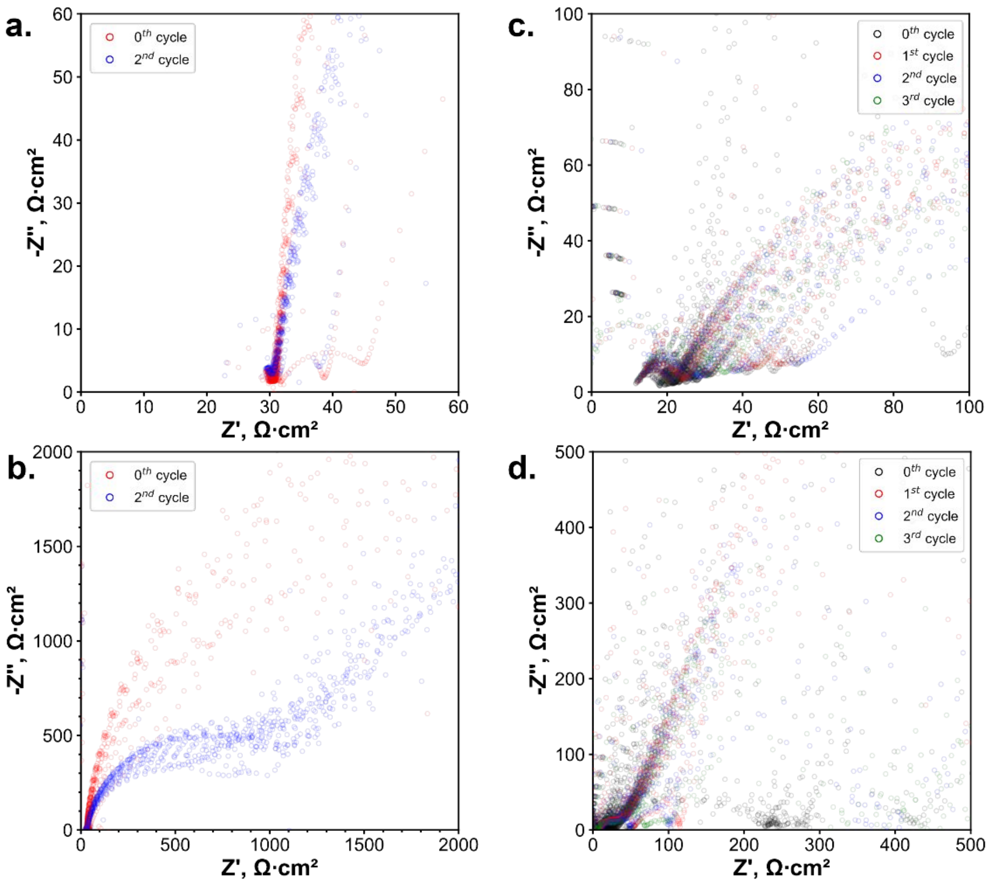

Figure S10. Nyquist plot of 36 EIS spectra for the unmasked substrate at high frequencies (a), at low frequencies (b); for the masked substrate at high frequencies (c), at low frequencies (d). Areal resistances were calculated based on the SDC tip area.

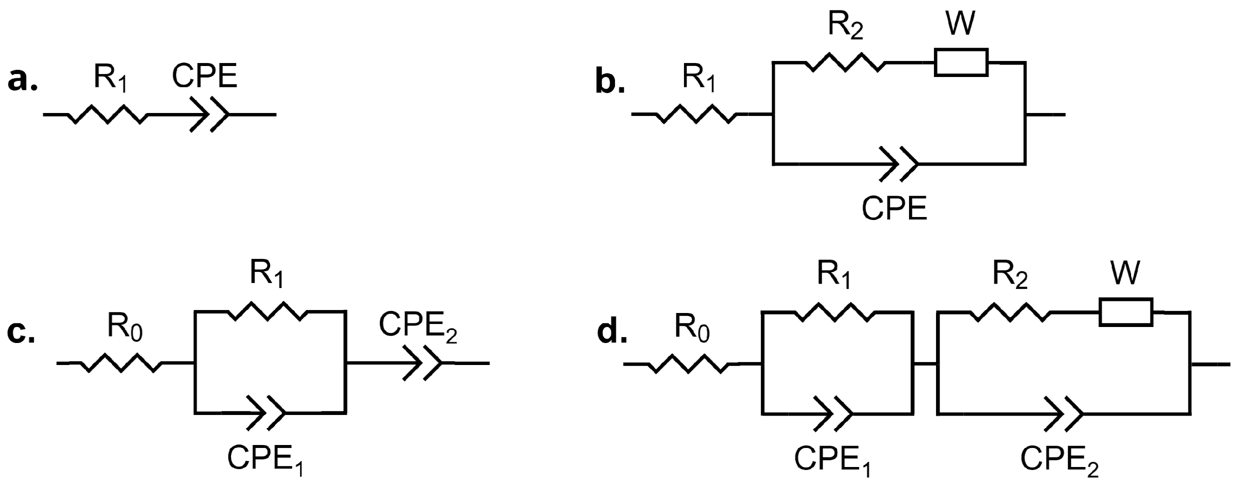

Figure S11. Equivalent circuits for 0th cycle on the substrate without a mask (a), 1st and 2nd cycle on the substrate without a mask (b): R_1 – corresponds to the sum of contact resistance and electrolyte resistance, R_2 – charge transfer and SEI resistance, W – Warburg impedance, CPE (constant phase element) indicates a capacitive response; for 0th cycle on the substrate with a mask (c), 1st, 2nd and 3rd cycle on the substrate with a mask (d): R_0 – corresponds to the contact resistance, R_1 – corresponds to the electrolyte resistance. R_2 – charge transfer and SEI resistance, W – Warburg impedance, CPE (constant phase element) indicates a capacitive response.

Statistical analysis

Table S1. Mean and 95%-confidence intervals for selected figures of merit

a.) Unmasked substrate

**Parameter**	**Mean ± 95% CI**
Q_{d1} [mAh/g]	230.6 ± 13.3
Q_{d2} [mAh/g]	243.8 ± 16.8
CE_1 [%]	39.9 ± 1.1
CE_2 [%]	69.8 ± 1.3
OCP_0 [V]	3.131 ± 0.060
OCP_2 [V]	0.525 ± 0.019
R_1 (0th cycle) [Ω·cm²]	30.5 ± 0.1
R_1 (2nd cycle) [Ω·cm²]	30.4 ± 0.6
R_2 (2nd cycle) [Ω·cm²]	1430 ± 149

b.) Masked substrate

**Parameter**	**Mean ± 95% CI**
Q_{d1} [mAh/g]	240.6 ± 6.8
Q_{d2} [mAh/g]	222.6 ± 3.2
Q_{d3} [mAh/g]	204.5 ± 2.9
CE_1 [%]	51.2 ± 0.5
CE_2 [%]	81.8 ± 0.6
CE_3 [%]	85.6 ± 1.0
OCP_0 [V]	3.278 ± 0.034
OCP_1 [V]	0.506 ± 0.011
OCP_2 [V]	0.479 ± 0.008
OCP_3 [V]	0.466 ± 0.008
R_0 (0th cycle) [Ω·cm²]	7.0 ± 1.7
R_0 (1st cycle) [Ω·cm²]	7.7 ± 1.4
R_0 (2nd cycle) [Ω·cm²]	10.1 ± 2.2
R_0 (3rd cycle) [Ω·cm²]	7.7 ± 1.6
R_1 (0th cycle) [Ω·cm²]	32.2 ± 8.7
R_1 (1st cycle) [Ω·cm²]	42.3 ± 27.7
R_1 (2nd cycle) [Ω·cm²]	41.2 ± 30.0
R_1 (3rd cycle) [Ω·cm²]	45.9 ± 34.2
R_2 (1st cycle) [Ω·cm²]	250.1 ± 23.0
R_2 (2nd cycle) [Ω·cm²]	252.3 ± 42.8
R_2 (3rd cycle) [Ω·cm²]	226.9 ± 24.4

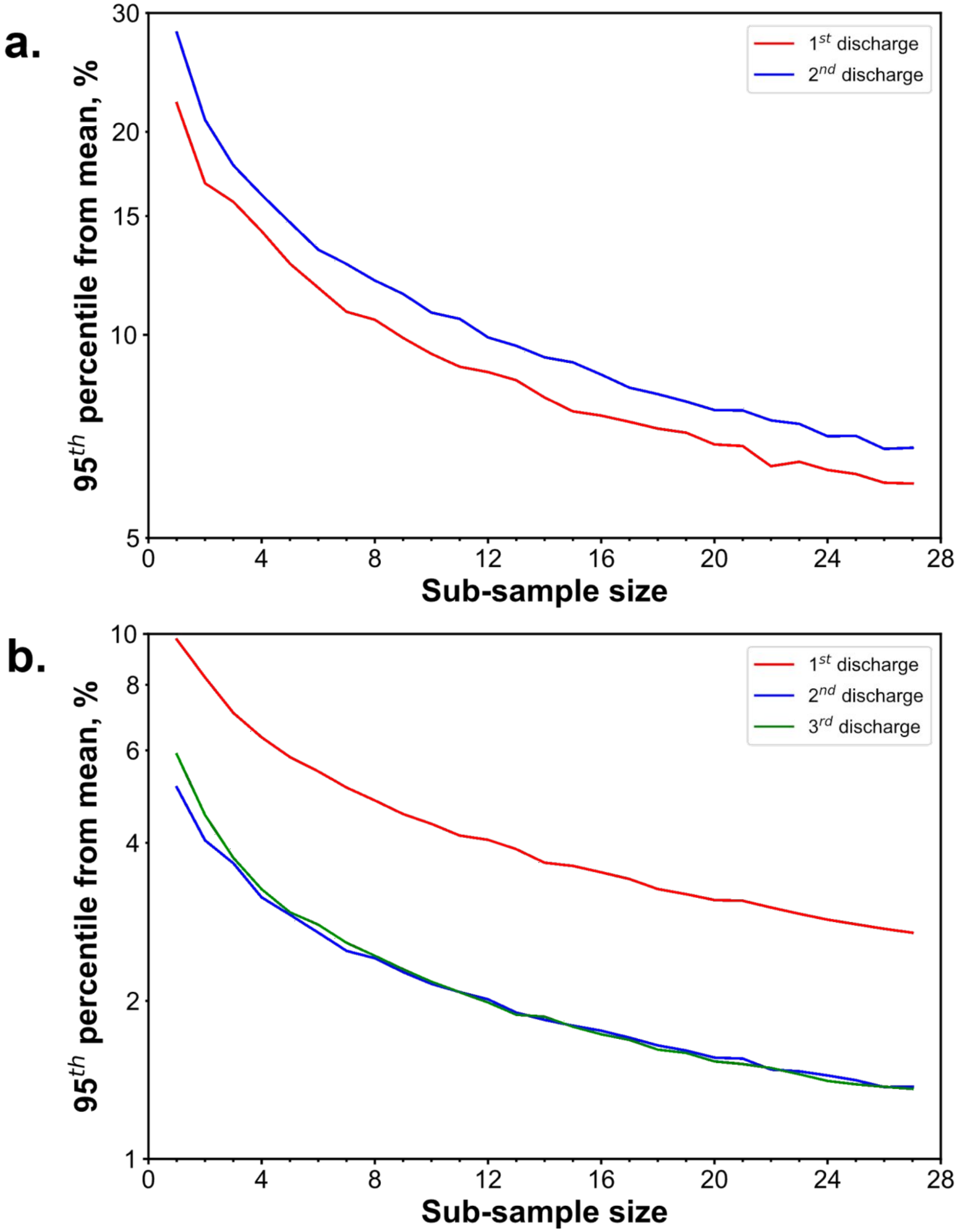

Figure S12. 95th percentile in percentage from the median of bootstrapped discharge capacity for different sub-sample sizes for the substrates without (a), with (b) masking.

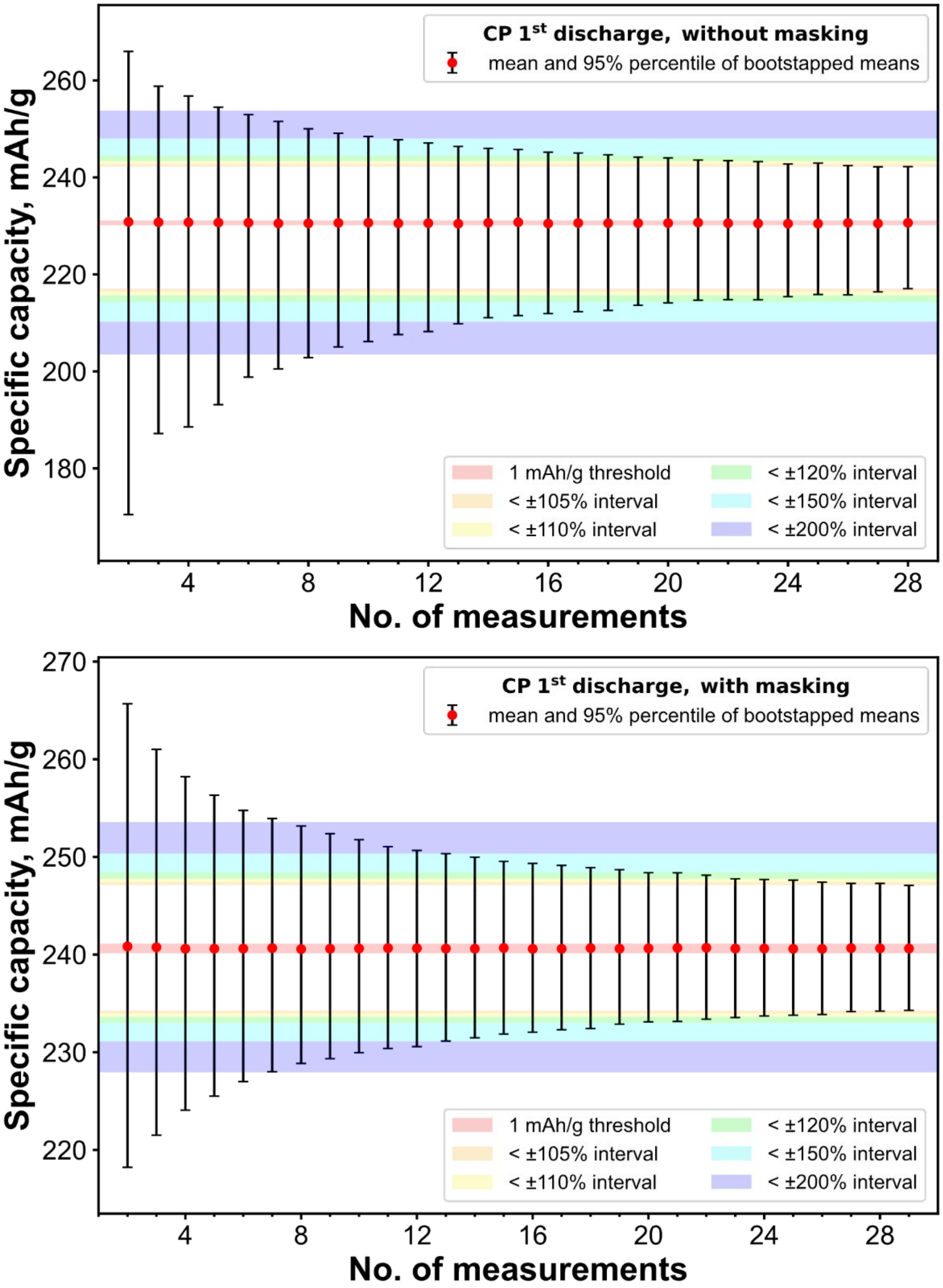

Figure S13. Sampling analysis of bootstrapped means: dependence of the mean and 95% percentile of the bootstrapped mean on the bootstrap sample size (number of randomly selected experiments during the resampling with replacement process, all successful experiments considered as a population) for the 1st discharge cycle for the substrate without (a), with (b) masking. All mean values of different bootstrap sample sizes are within ± 1 mAh/g threshold. The threshold for a minimum number of the required

experiments can be selected based on the difference between the bootstrap sample size and with bootstrap population size (in this case, < ± 200 % of the 95th percentile). 8 and 9 experiments are required to meet the desired statistical constraints for the unmasked and masked samples, respectively.
